# A wireless tattooing capsule endoscope using external electromagnetic actuation and chemical reaction pressure

**DOI:** 10.1371/journal.pone.0219740

**Published:** 2019-07-16

**Authors:** Manh Cuong Hoang, Viet Ha Le, Jayoung Kim, Eunpyo Choi, Byungjeon Kang, Jong-Oh Park, Chang-Sei Kim

**Affiliations:** 1 School of Mechanical Engineering, Chonnam National University, Gwangju, South Korea; 2 Medical Microrobot Center, Chonnam National University, Gwangju, South Korea; University of Colorado, Anschutz Medical Campus, UNITED STATES

## Abstract

In this paper, we present a tattooing capsule endoscope (TCE) that can localize an intestinal lesion or tumor for a preoperative laparoscopic surgery. The TCE is based on a wireless capsule endoscope (WCE) structure and can be actively controlled by an external electromagnetic actuation system to move, observe, and mark the target lesion in the gastrointestinal (GI) tract. The TCE is designed to perform capsule locomotion, needle extrusion and intrusion motions, and ink injection. First, the TCE is controlled to move to the target lesion during GI tract diagnosis via a capsule endoscopic camera. Further, a tattooing needle is extruded by an electromagnetically controlled mechanism to puncture the tissue. Finally, the tattooing ink is injected by the chemically reacted carbon dioxide gas pressure that is triggered by a shape memory alloy wire and a reed switch. The reed switch is also activated by the external magnetic field flux density. The suggested methods were verified by the ex-vivo experiments. The TCE prototype was able to move to the target lesion and inject the ink beneath the mucosa layer safely, thereby leaving a visible tattooed mark for surgical lesion identification. The proposed TCE method can accelerate the development of functionalities as well as tattooing procedures of the WCE in the GI tract.

## 1. Introduction

The gastrointestinal (GI) tract is an organ that suffers from many common and daunting diseases, particularly gastric cancer, which is the fourth most common cancer and the second leading cause of cancer-related deaths in the world [1–3]. The diagnosis and treatment of intestinal diseases are challenging for doctors because of the lack of visual information and invasive physical investigation such as palpation. Laparoscopic surgery is a minimally invasive surgery performed to treat digestive cancer, in which a segment of the bowel with ulcers is removed. The localization of lesions before the operation is a crucial step in laparoscopic surgeries. Inadequate identification of a lesion increases the surgery time and risk of resection of wrong segment of the bowel [4–6]. Therefore, endoscopic tattooing at colonic lesions is used as a safe and effective method for preoperative localization of clinical sites in laparoscopic surgeries. However, the endoscopic instrument can cause side effects, such as fear or sedation, in the patient, and may cause pain because of the stiffness of the endoscopic cable, which also limits its application in organs with sharp curvature geometry or deep positions. Therefore, a safe and practical methodology is required to enhance the tattooing procedure in the GI tract.

Nowadays, the cooperation between doctors and engineers and advancement within bioengineering creates significant development in healthcare technologies, from macro-scale [7,8] to micro- [9,10] and nano-scale [11,12]. As a promising alternative of endoscopy, a wireless capsule endoscope (WCE) has been developed and commercialized recently by several companies such as Given Imaging (Israel) [13], Jinshan (China) [14], and Intromedic (South Korea) [15]. In addition to diagnostic visualization, functionalities of the WCE have been widely studied to achieve various functional modules, such as biopsy, drug delivery, obesity treatment, and bleeding detection. Biopsy modules for WCE that can take sample tissues wirelessly have been presented by several research groups [16–18]. The WCE can be combined with a drug delivery module for the treatment of digestive diseases [19–22]. Furthermore, the WCE is also used for obesity treatment and bleeding detection [23,24]. These developments are based on the given WCE platform that utilizes the controllable untethered endoscopic capsule motions in the GI tract.

In this study, to address the limitations of conventional endoscopic tattooing and expanding clinical applications of the WCE, we present a WCE integrated with a tattooing module for marking the lesions, tumors, or polypectomy sites in the digestive organs. To the best of our knowledge, this is the first instance of integration of the tattooing function with the WCE, which has not been studied before because of various significant limitations, such as small size of the capsule body, ink injection force and pressure, and needle control.

The design specifications of the tattooing capsule endoscope (TCE) along with their functionalities are as follows. (1) External robotic manipulation is necessary to maximize the advantages of the WCE, which is required to move actively in the GI tract. To target a position in the digestive organs, the capsule should be able to steer the direction of the injection needle. The magnetic force from the external control system should be sufficient for the needle to penetrate the tissue. During locomotion, the injection mechanism should be disabled. Once the TCE reaches the target, it is triggered to inject ink beneath the mucosa layer. (2) For safety reasons, the capsule is capable of controlling the status of the needle. The tattooing needle should not be exposed outside the capsule body to avoid damage or perforation during locomotion. At the target, the capsule can actively extrude the needle to puncture the organ’s wall and retract after completing the tattooing process. (3) The injection mechanism should generate sufficient force to expel the tattooing ink from the container to the tissue. In this study, we focus on using a chemical reaction that can generate gas to push the piston, as the resultant force is controllable because of the content of the reactants. The gas-generation chemical reaction can produce a high pressure with a small amount of reacting substances. Therefore, we can optimize the size of the tattooing module as well as the capsule robot.

The result of these design specifications was a tattooing CE equipped with a chemical reaction-based injection module. The chemical reaction between two dry chemical powders was magnetically triggered by an electrical circuit that included a Shape-Memory-Alloy (SMA) wire and a reed switch. A needle control module (NCM) was also developed to ensure that the injection needle did not damage the overpassed organs. The suggested motions of the TCE were validated by ex-vivo experiments performed on five samples.

The remainder of this paper is organized as follows. We explain the components and working principle of the tattooing capsule endoscope (TCE) in detail in Section 2. We demonstrate and discuss the ex-vivo experimental results of the prototype TCE in Section 3. In addition, we also show how the different parameters of the TCE were designed. In Section 4, we discuss the conclusions, drawbacks of the TCE, and future works.

## 2. Materials and methods

### 2.1 Tattooing capsule endoscope design specifications

The TCE is a completely new technology of WCE. Therefore, to develop this miniature microrobot for medical applications, its design requirements are primarily determined considering clinical standards and functional constraints as follows.

#### Tattooing ink

Currently, the most common ink used in endoscopic tattooing is India ink because it lasts for a long time and has a low complication rate [25–27]. A tattoo with 0.2–0.5 ml aliquots of India ink and saline in 1:100 dilution rate can last as long as five months as a marker [27–29]. Therefore, in this study, we used 1:100 aliquot of India ink. The WCE is small in size; thus, we used a minimized amount of ink (0.2 ml); however, it is still visible, as illustrated in the experiments of the next section.

#### Tattooing position and injection depth

**Fig 1:**
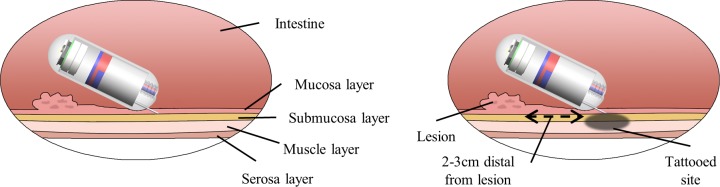
Recommended tattooing position. The right figure shows the injection in the submucosal layer with an oblique angle to the organ’s wall, and the left figure shows the injection location relative to the lesion margin.

Fig 1 shows the recommended position for marking the target in the digestive organs. The tattooing site should be around 2–3 cm distal from the anal or oral margin of the lesion [30,31]. To make it last long, diluted India ink should be injected beneath the mucosal layer (between the mucosal and submucosal layers) to create a visible bleb [25,27,30]. The thickness of the rectal mucosa layer was reported between 0.66 and 1.13 mm, while the total wall thickness was between 2.28 and 3.55 mm [32]. Furthermore, the needle was penetrated at an oblique angle to the organ wall to prevent the puncturing of the serosa [6]. Therefore, to inject the ink into the submucosal layer, a needle with 2 mm length was selected, and the insertion angle was less than 30°, which was expected to reach the stable submucosal layer (around 1 mm). The 2 mm needle was also reliable to avoid perforation of the digestive organs.

#### Injection needle

For actual implementation, a commercial medical needle should be used. The 26-gauge medical needle was chosen among several candidates (24–27 gauge medical needles). Considering the required force and pressure for the ink injection process, the experimental test shows that the most suitable needle was the 26-gauge type.

**Fig 2:**
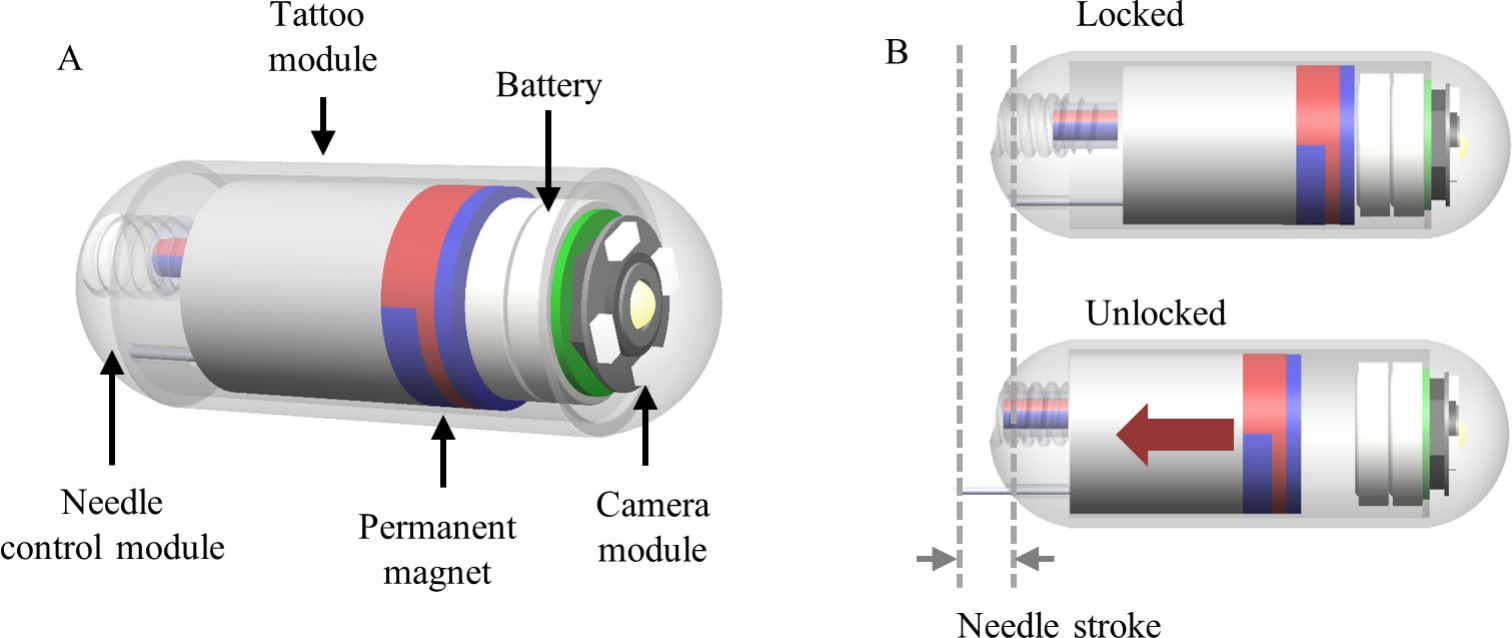
Schematics of the TCE. (A) Conceptual design of the WCE equipped with the tattoo module. (B) Two states of the NCM for needle extrusion.

Fig 2A shows the conceptual design of the proposed TCE with five main parts. A camera module powered by a battery is used to visualize the digestive organs. Two permanent magnets (PMs) with different magnetization direction are attached to the tattoo module (TM) for flexible locomotion and to control the status of the injection needle. The needle control module (NCM) acts as a key to lock and unlock the movement of the TM. Once the NCM is unlocked, the TM is movable along the axial axis of the capsule to expose and retract the injection needle, as shown in Fig 2B.

**Fig 3:**
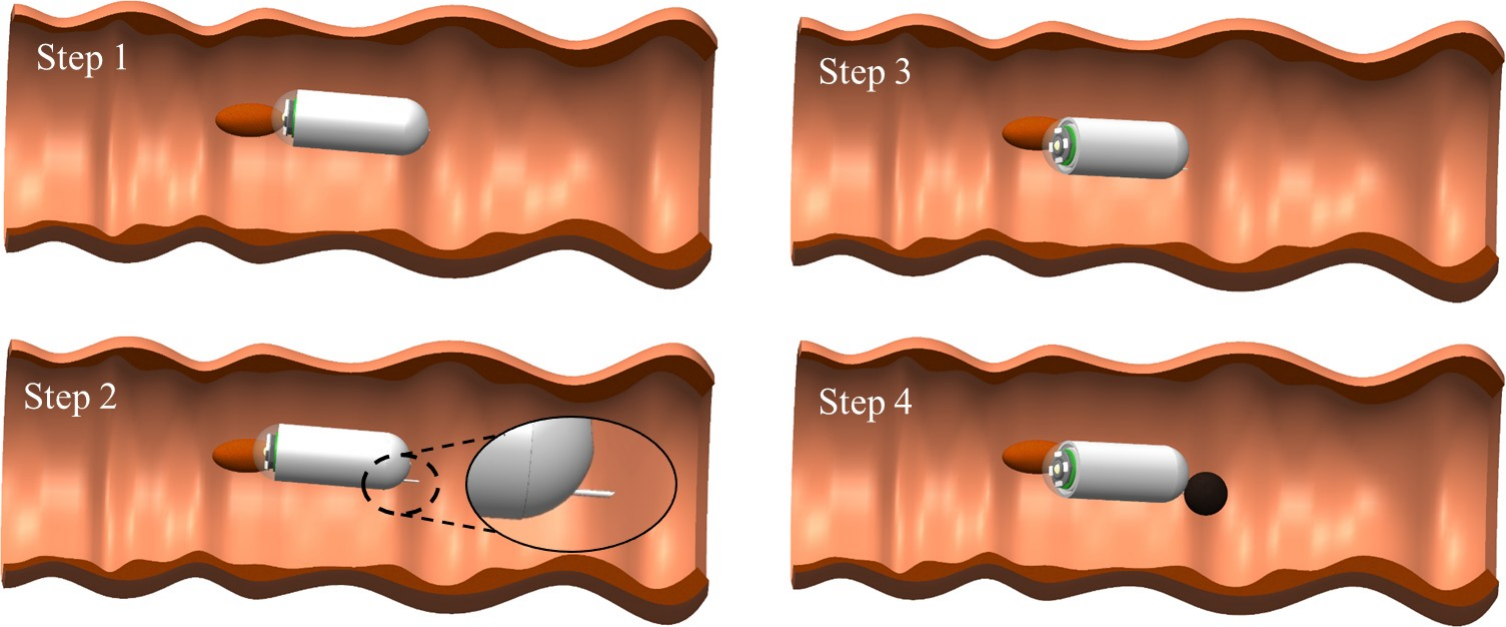
Tattooing procedure of the TCE controlled by the external magnetic field.

Fig 3 shows the whole sequence of the tattooing procedure. In the first step, after the patient swallows the TCE, the EMA system is powered to drive it for scanning the digestive organs. In the second step, the injection needle is extruded out once the TCE reaches the target (lesion, tumors, or biopsied site). Thirdly, the operator controls the TCE’s orientation to insert the needle into the submucosal layer at an oblique angle to the intestinal wall to avoid wrong needle penetration or cause perforation. Finally, the chemical reaction is activated to inject the ink into the submucosal space, creating a black visible bleb. The needle is then retracted back inside the TCE’s body for moving out safely.

### 2.2 Tattooing module

**Fig 4:**
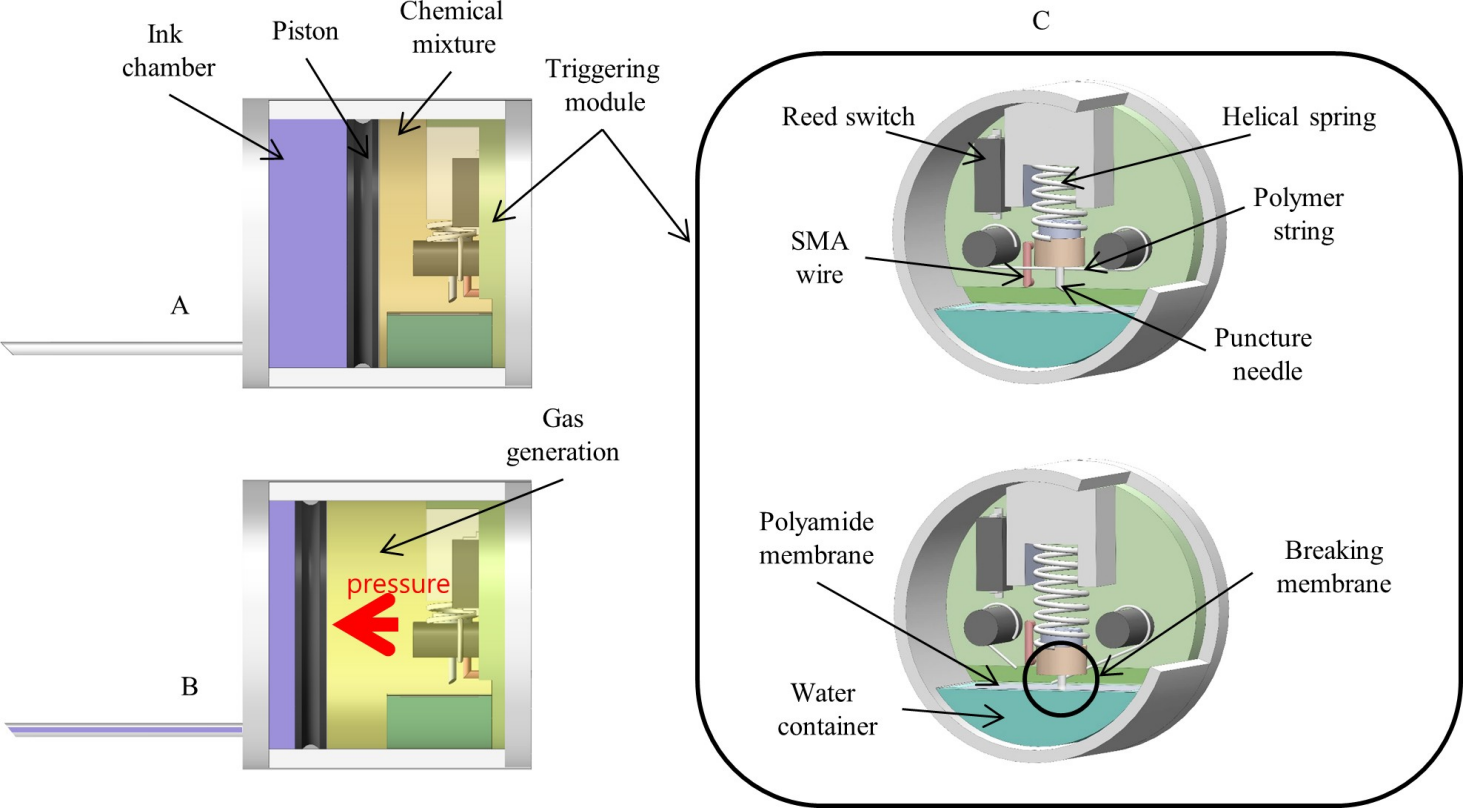
Tattooing module in details. (A) Tattooing module with components at the initial state. (B) Tattooing module after triggering the chemical reaction. (C) The chemical-reaction triggering module using reed switch.

Tattooing module is a capsule-independent body utilizing a propellant gas from the chemical phenomenon to inject ink into submucosal layer. It is composed of two compartments separated by a rubber piston, as shown in Fig 4A. The ink chamber contains 0.2 ml of tattooing ink and the propellant room contains a dry chemical mixture (sodium phosphate monobasic (SPM) and sodium bicarbonate (SB) in dry powder form). Once the mixture comes in contact with water, a chemical reaction occurs and produces carbon dioxide (CO_2_). The TM is a closed chamber; thus, the generated gas increases the pressure inside the propellant room. When the pressure reaches the threshold, the resultant force exerted on the piston is sufficient to make it move and eject the ink out, as shown in Fig 4B. To activate ink injection, we designed a module to trigger actively the gas-generation reaction. Fig 4C shows the internal mechanism of the chemical-reaction triggering module. The water container is made of an open-end 3D-printed part with a volume of 0.02 ml. The open end is sealed with a polyamide membrane, which is inert to reagents and their reaction solutions, to separate the water and chemical powders. To trigger the reaction, we need to break the sealed membrane and make contact between water and the reagents. A sharp puncture needle is attached at one end of a helical spring while the other is fixed. Initially, the needle is held 1 mm above the polyamide membrane by a polymer string so that the stored potential energy is sufficient to break the membrane during release. An SMA wire connected in series with the batteries via a reed switch is used to cut the polymer string by heat. A reed switch is an electrical component that can switched to another state by a certain pull-in value of the magnetic field intensity. Therefore, by using the electromagnetic control system, we are able to control the state of the reed switch wirelessly. When the reed switch is turned on, the SMA wire gets heated up and cuts the string. Consequently, the puncture needle is pushed down and the sealed membrane is punctured.

### 2.3 Needle control module

**Fig 5:**
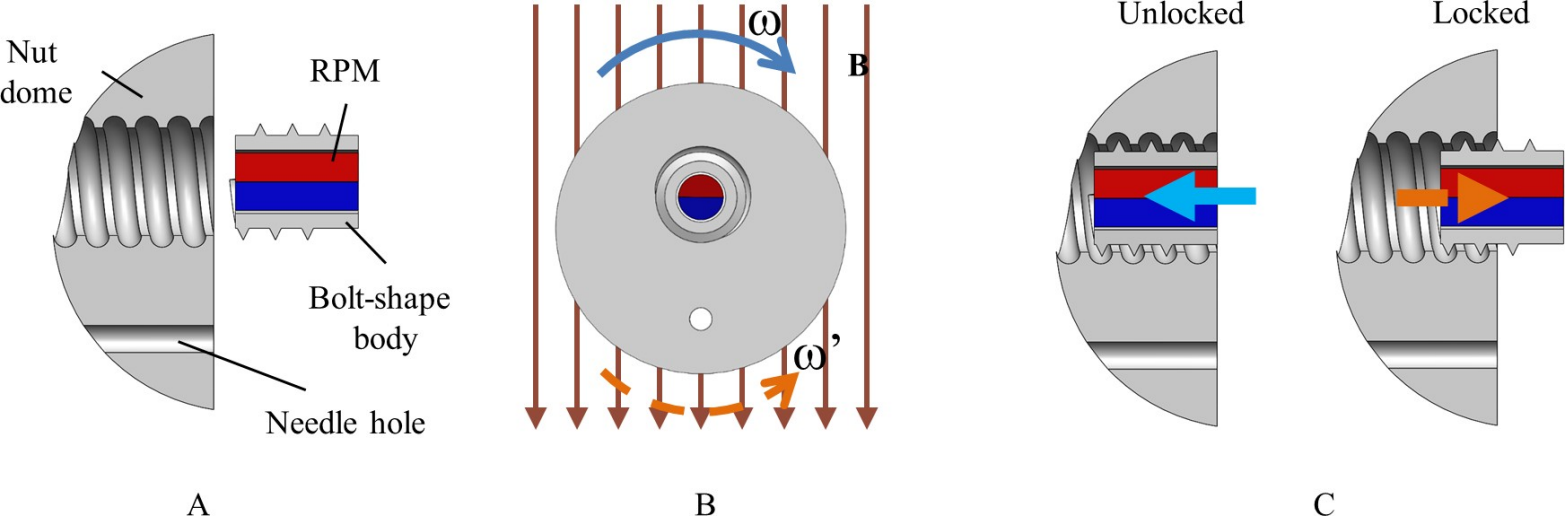
Needle control module. (A) NCM components. (B) Rotating magnetic field rotates the permanent magnet. (C) Rotating motion of the RPM is converted into translation motion to switch the status of the NCM.

The needle control module is used to extrude and retract the tattooing needle by unlocking the translational motion of the TM. Fig 5A shows the structure of NCM, which is composed of three components, i.e., rotational permanent magnet (RPM), bolt-shape body, and nut dome. The RPM is 5 mm long and 2 mm in diameter with radial magnetization. The bolt-shape body with three revolutions of 1.1 mm pitch covers the RPM. Initially, the NCM is in a locked state. Therefore, the TM is blocked by the RPM, as shown in Fig 2B. The PM interacts with the generated non-rotating magnetic field for locomotion without undesirable extrusion of the needle. At the target location, the rotating magnetic field with ω direction is applied to rotate the RPM, and the bolt-shape body converts the rotation motion of the RPM into translation motion with the help of nut dome. The NCM is then unlocked, as shown in Fig 5C. Unlocking the NCM enables the TM to slide inside the capsule’s body. The operator can control the magnetic and gradient fields to push the PM backward. Consequently, the needle is extruded out, as shown in Fig 2B. As the tattooing process is complete, the needle is retracted back inside the capsule body to prevent damage while moving out. The operator pulls the PM back to withdraw the needle. The rotating magnetic field with reverse direction creates a torque rotating RPM to lock the NCM again, thereby disabling the movement of the TM (see Fig 2B). When the NCM is in the locked state, the PM performs the locomotion function.

### 2.4 TCE manipulation method

**Fig 6:**
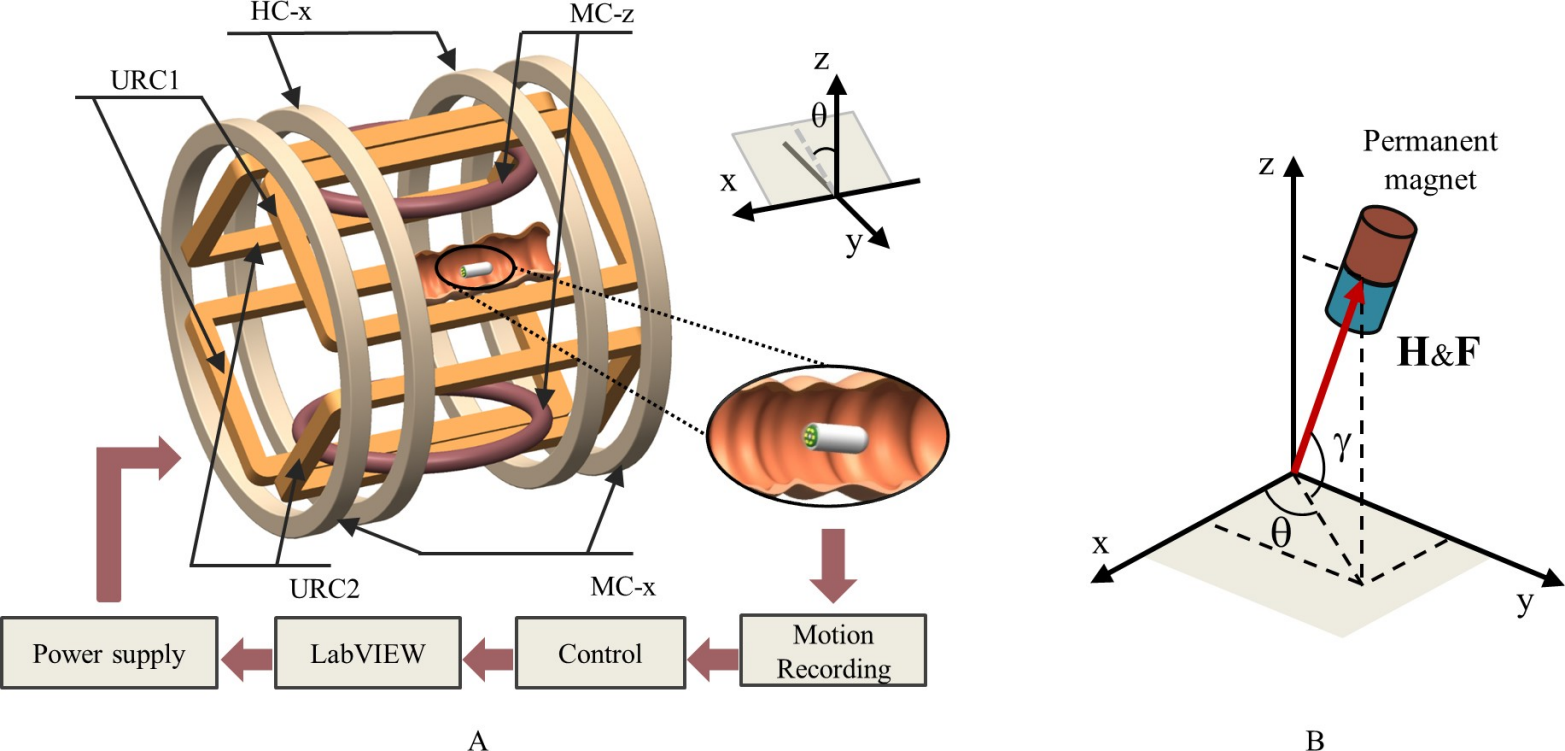
EMA system for capsule manipulation. **(A) Configuration and control scheme of the EMA system. (B) Actuation mechanism of the TCE; the arrow indicates the generated magnetic field vector** H **and force vector** F.

The TCE takes advantage of the untethered WCE; thus, a wireless remote-control method is essential for its practical implementation. We designed an EMA system that can generate strong magnetic and gradient fields to control the functional CE. Fig 6A shows the schematic configuration and control scheme of the custom-designed EMA system. The control method from our previous system, ALICE, is applied to this system [25]. A Helmholtz coil and two custom-designed uniform rectangular coils (URC1 and URC2) are used to generate a uniform magnetic field (UMF) for capsule alignment. Furthermore, two Maxwell coils are used to create a gradient magnetic field (GMF) overlaying the UMF to propel the capsule. To enhance the magnetic and gradient fields, the number of turns of the coil are increased, as presented in Table 1.

**Table 1:** Technical specifications of the EMA system.

Coils	HC-x	MC-x	MC-z	URC1, 2
**Radius (mm)**	195	195	100	n/a
**Width × length (mm)**	n/a	n/a	n/a	156 **×** 337
**Distance (mm)**	195	337	173	200
**Number of turns**	710	1426	660	600

When the electromagnets are powered, the generated magnetic field exerts a torque **τ** and a force **F** on the permanent magnet in a region of interest (ROI), which can be computed as follows:

$$\tau =\mu_{0}VM\times H$$ (1)

$$F=\mu_{0}V(M\cdot \nabla )H=\mu_{0}V{\left[\begin{array}{ccc} \frac{\partial H}{\partial x} & \frac{\partial H}{\partial y} & \frac{\partial H}{\partial z} \end{array}\right]}^{T}M$$ (2)

where *μ*_0_ = 4π ×10^−7^ T·m/A, **H** is the generated magnetic field vector, and *V* and **M** are the volume unit and magnetization vector of the permanent magnet, respectively [33,34]. Fig 6B shows the actuation mechanism of the TCE with a permanent magnet using the external magnetic field, where the magnetic force direction is set identical to that of the UMF.

The UMF, which aligns the magnet toward the planned direction, can be described as follows:

$$H=\left[\begin{array}{c} Hx\\ Hy\\ Hz \end{array}\right]=\left[\begin{array}{c} H\;\cos\gamma \cos\theta \\ H\;\cos\gamma \sin\theta \\ H\;\sin\theta \end{array}\right]$$ (3)

where H, *γ*, and *θ* are the desired values of magnitude, pitching angle in degrees, and yawing angle in degrees of the UMF vector, respectively. The UMF inside the ROI is the summation of the magnetic field produced by HC-x, URC1, and URC2 because of the electromagnetic field’s superposition property. The magnetic field components in the Cartesian coordinate of HC-x in the ROI can be calculated as follows [33]:

$$H_{H}={\left[0.7155\frac{i_{H}\times n_{H}}{r_{H}}\;0\;0\right]}^{T}$$ (4)

where *i*_*H*_, *n*_*H*_, and *r*_*H*_ are the input current, number of copper coil turns, and radius of the Helmholtz coil, respectively. The magnetic field in the ROIs of URC1 and URC2 can be described as follows:

$$H_{URC}={\left[0\;\pm 0.264\frac{i_{U}\times n_{U}}{d_{U}}\;0.264\frac{i_{U}\times n_{U}}{d_{U}}\right]}^{T}$$ (5)

where *i*_*U*_, *n*_*U*_, and *d*_*U*_ are the input current, number of copper coil turns, and width of the URC coil, respectively. The ± sign corresponds to URC1 and URC2, respectively. From (3), (4), and (5), we can obtain the input currents for HC-x, URC1, and URC2 to generate the desired UMF by solving the following equation:

$$H=H_{H}+H_{URC}$$ (6)

The GMF required to generate the propulsion force are obtained as follows:

$$F=\left[\begin{array}{c} F_{X}\\ F_{Y}\\ F_{Z} \end{array}\right]=\left[\begin{array}{c} F\;\cos\gamma \cos\theta \\ F\;\cos\gamma \sin\theta \\ F\;\sin\theta \end{array}\right]$$ (7)

where F is the magnitude of the desired force and *γ* and *θ* are the driving direction angles in degrees given by an operator. Furthermore, the GMF produced using the Maxwell coils can be calculated as follows:

$$H_{M}={\left[g_{M}x\;-0.5g_{M}y\;-0.5g_{M}z\right]}^{T}$$ (8)

$$g_{M}=0.6413\frac{i_{M}\times n_{M}}{r_{M}^{2}}$$

where *i*_*M*_, *n*_*M*_, and *r*_*M*_ are the input current, number of copper coil turns, and coil radius of the Maxwell coils, respectively [33]. From (2), (7), and (8), we can obtain the input current for the two Maxwell coils from the desired magnetic force vector given by a clinician or operator.

### 2.5 In-vitro and ex-vivo experiments preparation

**Fig 7:**
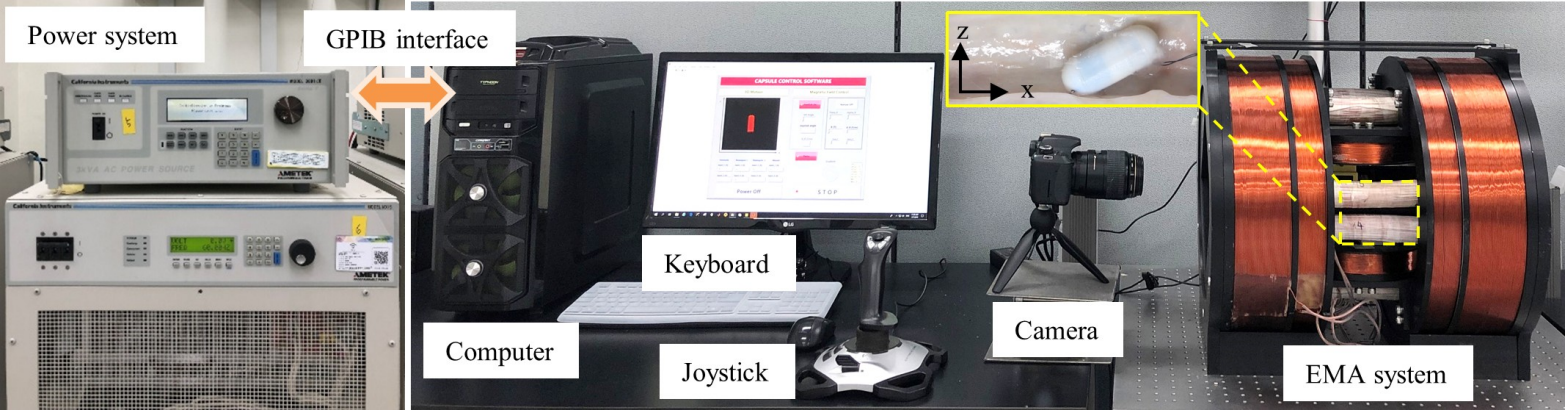
Real experiment setup for the in-vitro and ex-vivo tests.

For the validation of the proposed method, we conducted both in-vitro and ex-vivo tests. The real experiment setup is shown in Fig 7. The capsule was aligned by the magnetic field and pushed by the magnetic force where their magnitudes (H, F) were entered using a keyboard and the direction parameters (*γ* and *θ*) were set by a joystick (Extreme 3D Pro Joystick, Logitech). The controlled parameters were inputs of the control software built in LabVIEW 2017 (National Instruments) to compute the electrical currents of each electromagnet. The power supplies (MX15 2 units and 3001LX 3 units from California Instrument) were connected to a computer and controlled by PCI-based GPIB controller to provide current to each coil using the control command via the software. A small camera (C930, Logitech) was mounted inside the system to have a top view (xy plane) of the motion of the capsule. We used another camera (Canon, 600D), placed outside the system, to record the movements of the robot in the xz plane.

In the in-vitro validation, a 2D phantom model of the stomach was fabricated using VeroWhite material and placed in the ROI within the xy plane. In this test, we ignored the friction phenomenon because the purpose was testing the locomotion of the capsule within a non-tubular environment and verifying the triggering performance of the reed switch.

In the ex-vivo validation, a fresh pig’s small intestines (middle section) purchased from Homeplus supermarket, Donggwangju, Buk-ku, Gwangju, South Korea, (geographic coordinates: 35.179497(N) and 126.930365(E)) were prepared. First, the intestine was cleaned. Further, it was fixed on a semi-tube (20 mm in diameter and 100 mm in length) at the top and bottom edges so that the intestine was still elastic and flexible. In other words, the intestine could be moved flexibly according to the movements of the capsule.

## 3. Results and discussion

### 3.1 TCE prototype

**Fig 8:**
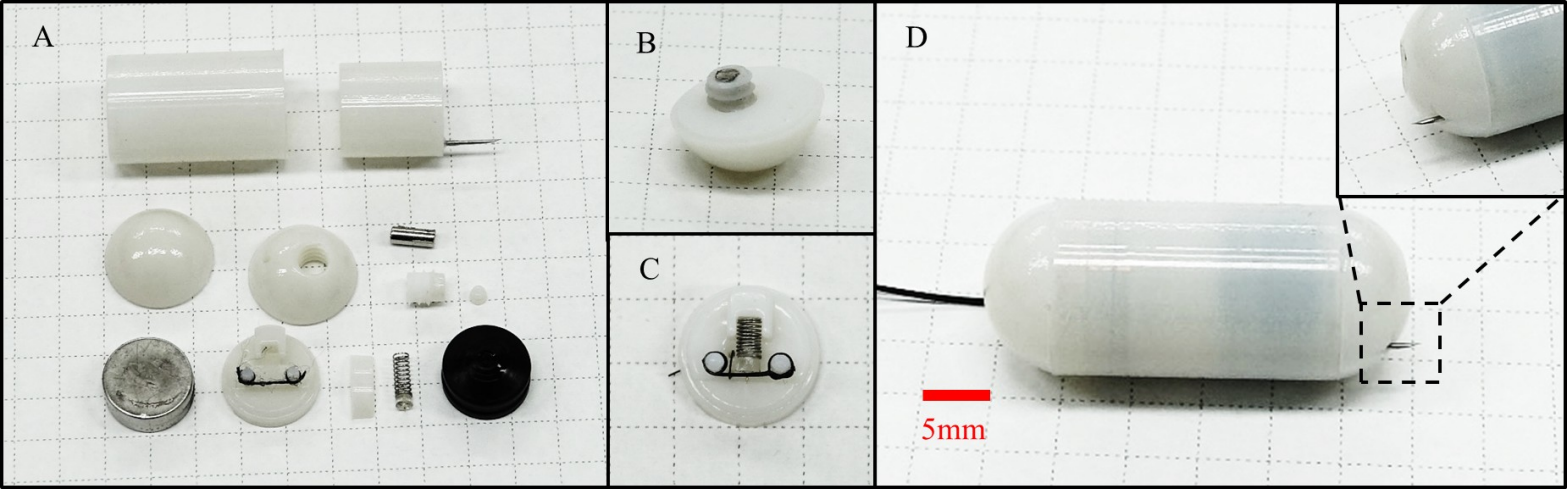
TCE fabrication. (A) Components of the TCE. (B) Assembled NCM. (C) Assembled triggering module. (D) Assembled TCE prototype.

The TCE prototype was manufactured using a rapid prototyping method and commercial components. Fig 8A shows the assembling components of this prototype. A cylindrical permanent magnet (5 mm in radius and 4 mm in thickness) and a helical spring with 0.5 N/mm stiffness (1 mm in radius and 5 mm in length) were used (MISUMI Co., South Korea). A rubber piston was obtained from a 3 ml syringe that had a diameter of 9.7 mm and height of 6 mm. We used a commercially available injection with 26-gauge medical needle (CPL CO. LTD, South Korea). The outer shell and TM body were printed by a 3D printer (Eden260VS, Stratasys Direct Manufacturing Ltd, USA) with VeroWhite material. The thicknesses of the shell and TM body were 0.6 mm each and the gap between them for sliding was 0.2 mm. Fig 8B and Fig 8C shows the assembled needle extrusion module and the triggering module, respectively. Finally, all sub-modules were assembled, as shown in Fig 8D. The prototype was 12.4 mm in diameter and 30 mm in length.

### 3.2 Tattooing module evaluation

**Fig 9:**
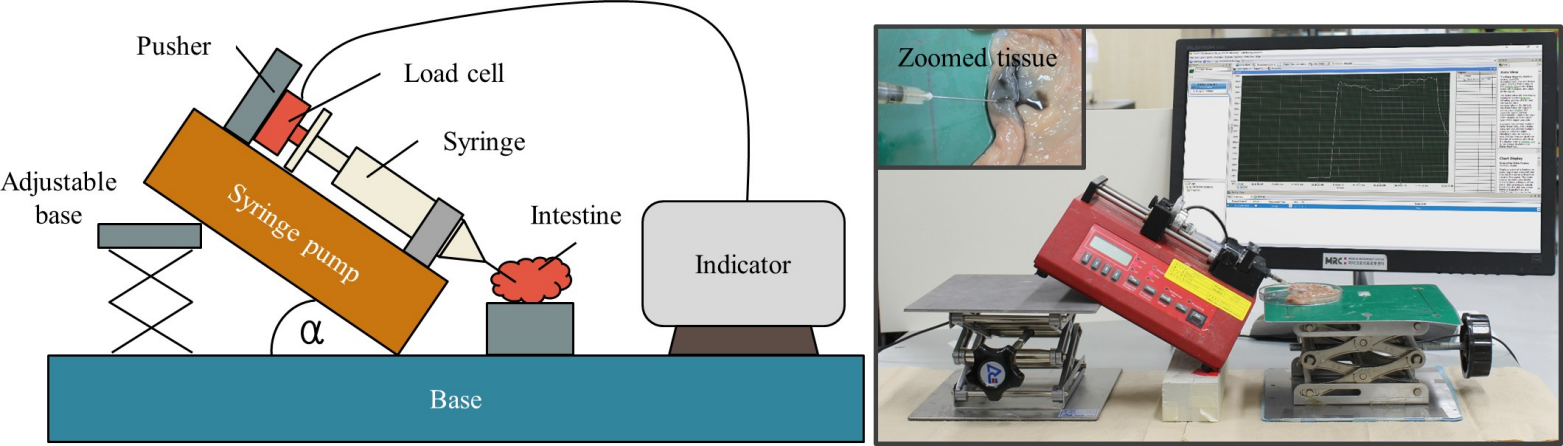
Experiment to evaluate the submucosal injection force. The left figure is the schematic diagram of the experiment setup and the right figure shows the actual photoshoot.

**Fig 10:**
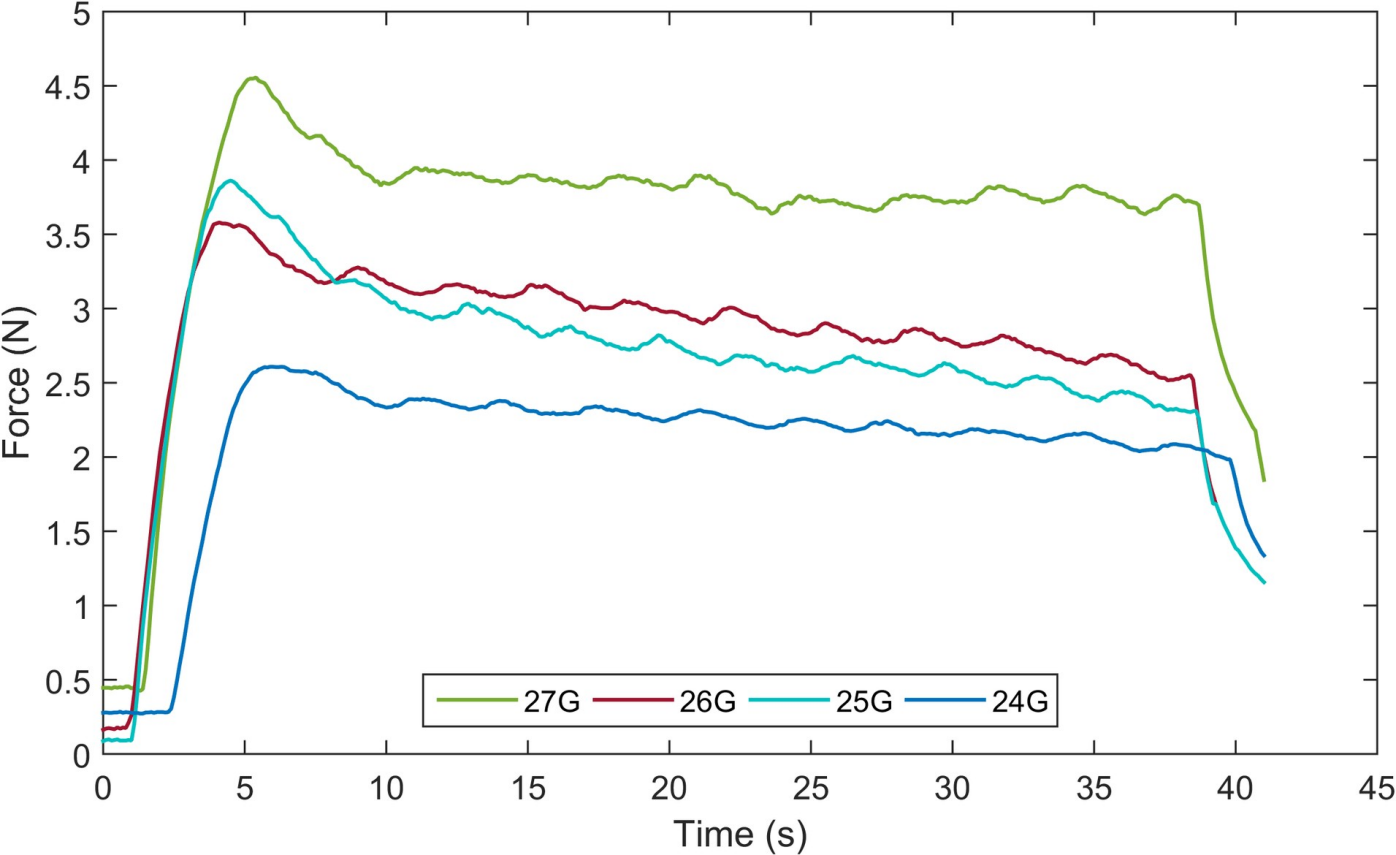
Experimental result of the submucosal injection force with needles of various sizes.

The force required to push the piston for ink injection was generated by carbon dioxide gas pressure. Therefore, to know the amounts of necessary reagents, firstly we need to know the required force to deliver the ink into submucosal layer. Instead of numerical computation, we conducted an experiment to investigate the injection force into the submucosal layer of the pig’s intestine (see Fig 9). The experiment was conducted with needles of various sizes, i.e., 24, 25, 26, and 27 gauge. A 3 ml syringe was used because it had the same inner diameter as that of the TM. The NE-1000 Programmable Single Syringe Pump manufactured by New Era Pump Systems was used to eject the ink into the submucosal layer. The syringe pump is fixed inclined at an angle α = 30° with respect to the horizontal plane. A GSO series load cell with a capacity 1000 g manufactured by Transducer Techniques was attached to the pusher part of the syringe pump. The load cell was connected to an amplifier TMO-2 and then calibrated to have linear characteristic. Furthermore, 0.2 ml of India ink with 1:100 dilution was loaded in the syringe and pumped with a pumping rate of 0.6 ml/min. The experiment was conducted at room temperature. Fig 10 shows the experimental results of the ink injection force with a sampling rate of 1 kHz. Due to the static friction force between the piston and syringe, the injection force reaches to a peak, then reduces gradually, and finally drops dramatically with the decrease of ink volume in the syringe. A high force (approximately 4.5 N) is required to expel the fluid using a 27-gauge needle, which requires more reagents and loading space. This value is significant lower (about 2.5 N) for a 24-gauge needle; however, the size of this needle is not advantageous for the penetration force. The 25- and 26-gauge needles show a similar result because they have the same inner diameter. However, the 26-gauge needle is the most suitable one because of its small outer diameter (0.46 mm). We can see that to win against the static friction force, we need more than 3.5 N injection force. Once the piston is moving, the required injection force reduces because of the kinetic friction force and the reducing ink volume. Therefore, we set the required threshold of pushing force at 3.5 N to inject the ink into the submucosal layer.

**Fig 11:**
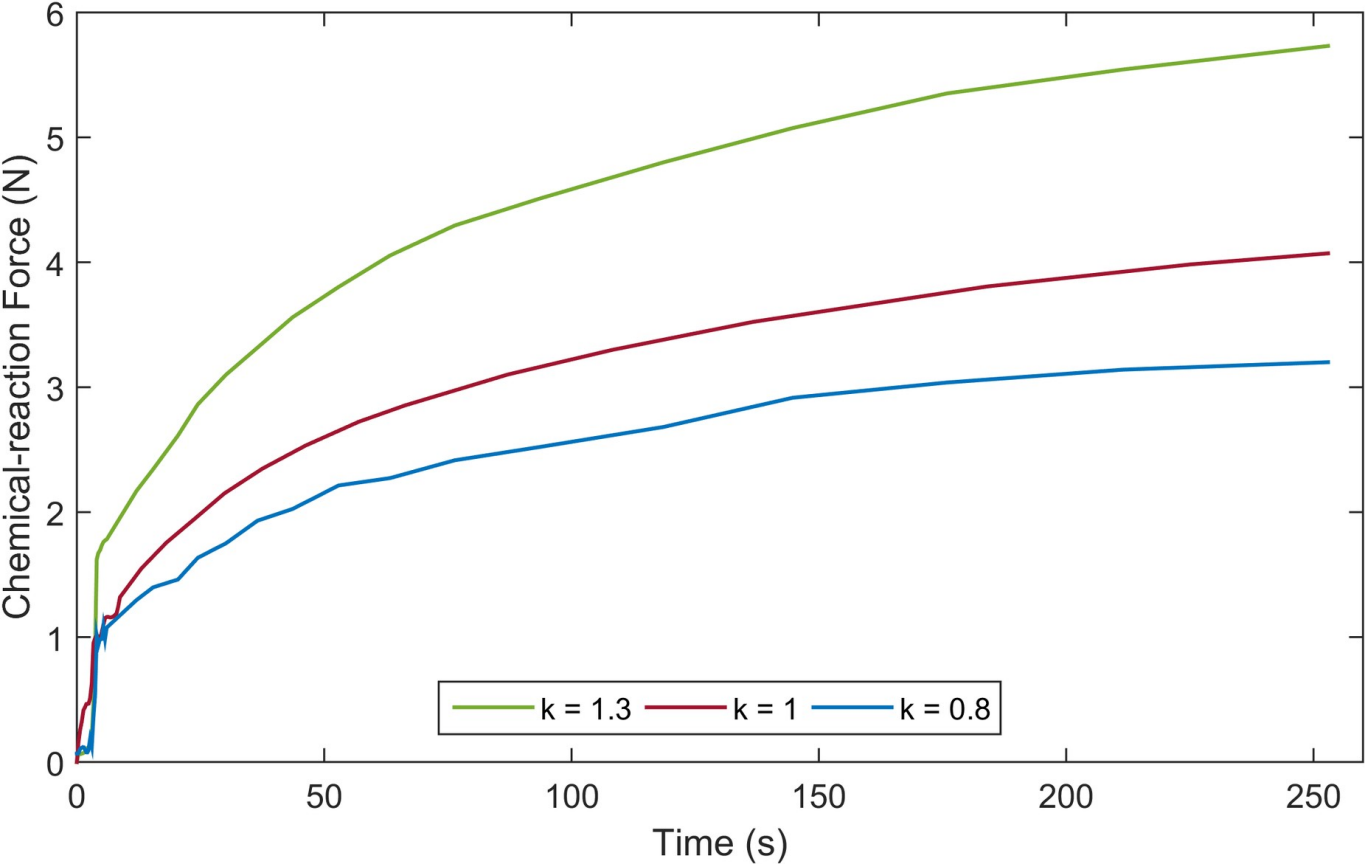
Experimental results of the chemical reaction force for various amounts of reactants.

After quantifying the required ink-injection force, we estimated the necessary amounts of reagents using ideal gas law. The solution is affected by several factors, such as friction, solubility, temperature, and even heat of formulation; thus, it is necessary to verify the resultant force corresponding to the increase in the generated gas. The calculated values of the compounds were used to derive various amounts of reagents with multiplier k = 0.8, 1, and 1.3 to examine the propellant force. We conducted an experiment similar to the previous one to determine the optimal amounts of the reactants (SB and SPM). The intestine and injection needle were removed from the previous experiment. A rubber piston was used to seal one end of the 3 ml syringe; the other along with a movable piston was connected to the load cell to measure the generated force. Further, 0.02 ml of water was added through the sealing piston via a 30-gauge needle syringe to activate the chemical reaction. The measured results were recorded for five minutes with a sampling rate of 1 kHz. Fig 11 shows the real-time propellant force with different amounts of reagents. Based on these experimental results, we decided to use 0.105 mg of SB and 0.15 mg of SPM (k = 1), which creates 3.5 N after 150 seconds.

### 3.3 Electromagnetic force evaluation

**Fig 12:**
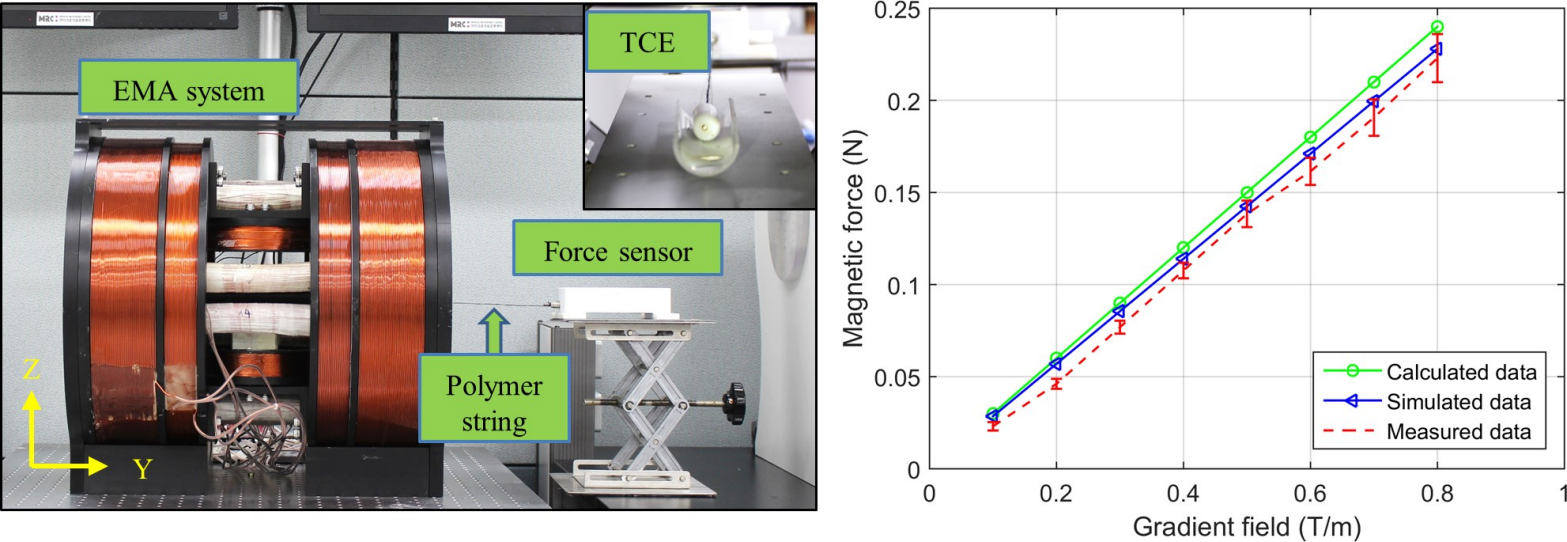
Measurements of magnetic force exerted on the TCE. (A) Experimental setup. (B) Electromagnetic force exerting on the TCE by the EMA system at various levels of the gradient field.

In the proposed TCE system, the external electromagnetic field plays an important role to generate the capsule driving force, needle extraction and retraction force, and needle penetration motion. We conducted an experiment to examine the electromagnetic force, as shown in Fig 12A. A force sensor (Advanced Digital Force Gauges Series 5, Mark-10) was connected to the TCE placed inside the ROI of the EMA system. The sensor was fixed outside the control system to prevent the effect of the produced magnetic field. A 10 mT magnetic field was generated to align the capsule along with the y axis and then the GMF was applied to create the pulling force in the -y direction. The tension of the string was equal to the pulling force; thus, the tension measurements was sampled continuously at sampling rate of 1 kHz. Fig 12B shows the comparison of the magnetic force at various gradient field levels from the experimental, theoretical, and numerical simulation obtained using the COMSOL Multiphysics software. The experimental peak value was 0.22 N, which was sufficient to move TCE in digestive tract and puncture the injection needle through the mucosal layer.

### 3.4 Triggering module verification

**Fig 13:**
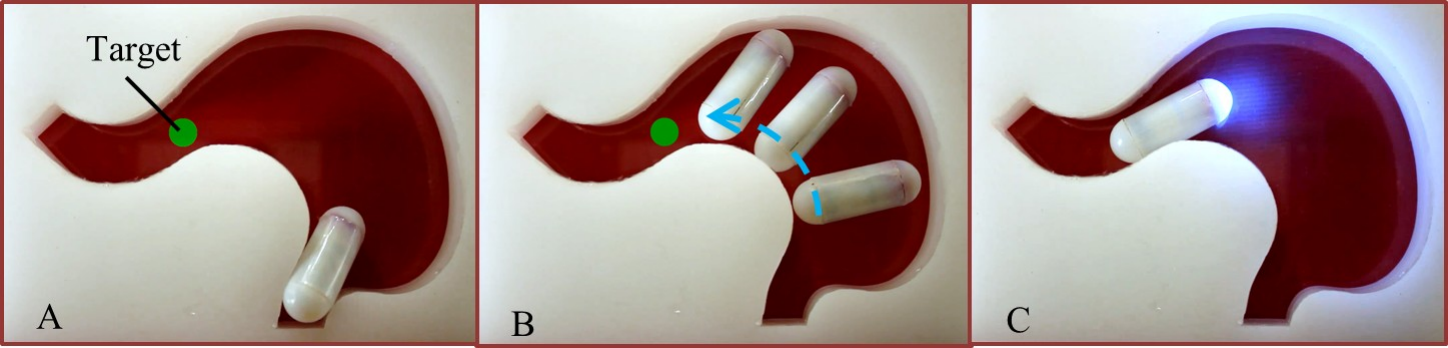
Reed switch controlled by EMA verification. (A) Starting position of the capsule with LED OFF. (B) Overlapped picture of the capsule’s movement while maintaining the LED state. (C) Capsule reaches the target and the reed switch is activated, thereby turning LED ON.

To verify the performance of the reed switch controlled by the external electromagnetic field, an experiment was executed to test the excitation level. The reed switch MK24-E-3 (Standex MEDER Electronics) is a normally open (NO) contact type with an ultra-miniature surface mount of 5.5 mm in length and 2.5 mm in height. Its typical magnetic sensitivity is in the range of 2.5–3 mT. The pull-in value was very sensitive to the operating magnetic field, approximately up to 30 mT within the ROI; thus, we covered the reed switch with the Mu-metal sheet to lower its sensitivity. A small LED was used to observe the status of the reed switch by connecting to batteries in series via the NO reed switch. If the switch was activated, the LED would turned on and vice versa. The whole circuit was installed inside the capsule prototype. First, we placed the capsule in the ROI of the EMA system and applied magnetic field (without gradient field) incrementally to determine the excitation value of the covered reed switch. When the magnetic field reached 46 mT, the LED was ON. Therefore, 46 mT is the excitation threshold of the shielded reed switch. To verify the performance of the triggering module during locomotion, the capsule was controlled in a 2D phantom stomach, as shown in Fig 13A. The main objective of the operator was to drive the capsule toward the target without turning the LED ON and powering it ON at the destination. Initially, the capsule started in the OFF state of the LED, which meant that the reed switch was not activated. The experimental excitation value of the reed switch was set at 46 mT; thus, the applied uniform and gradient magnetic fields for the locomotion of the capsule were limited to 10 mT and 800 mT/m, respectively. As shown in Fig 13B, the capsule could move to the target safely without triggering the reed switch. At the desired position, the gradient field was removed, and the magnetic field was increased to 50 mT to turn the LED ON (see Fig 13C). We could confirm the feasibility and reliability of the wireless activation approach by reed switch in the experimental results.

### 3.5 Ex-vivo experiments

**Fig 14:**
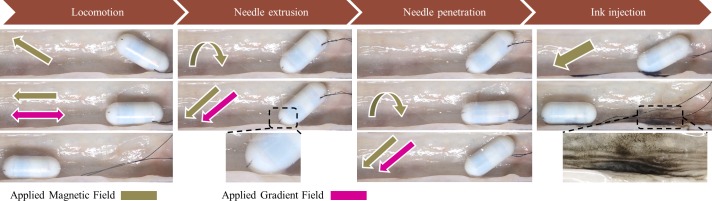
Ex-vivo experimental results conducted in pig’s small intestine with four steps of the TCE procedure. The arrow shows the direction of the vector; the straight arrow represents the non-rotating magnetic field and the rotation arrow represents the rotating magnetic field.

We performed ex-vivo experiments for TCE evaluation in a segment of the pig’s small intestine described in Section 2. Fig 14 shows the four main steps to mark the target. In the locomotion step, the capsule was aligned with the magnetic field and then the gradient field was applied in the same/reverse direction as that of the magnetic field to push the capsule forward/backward. After reaching to the target, 5 mT rotational magnetic field was created to rotate the RPM and unlock the NCM. The magnetic and gradient fields were then produced in the same direction to translate the TM and extrude the injection needle. Further, based on the position of the target (top, bottom, or side), the capsule was steered by the rotating magnetic field (10 mT) to direct the injection needle toward the target. Simultaneously, 800 mT/m of the gradient field was applied to penetrate the needle into the tissue. Finally, the magnetic field was increased to 50 mT to close the reed switch and trigger the chemical reaction-based ink injection. As the tattoo procedure finished, the capsule was driven out.

**Fig 15:**
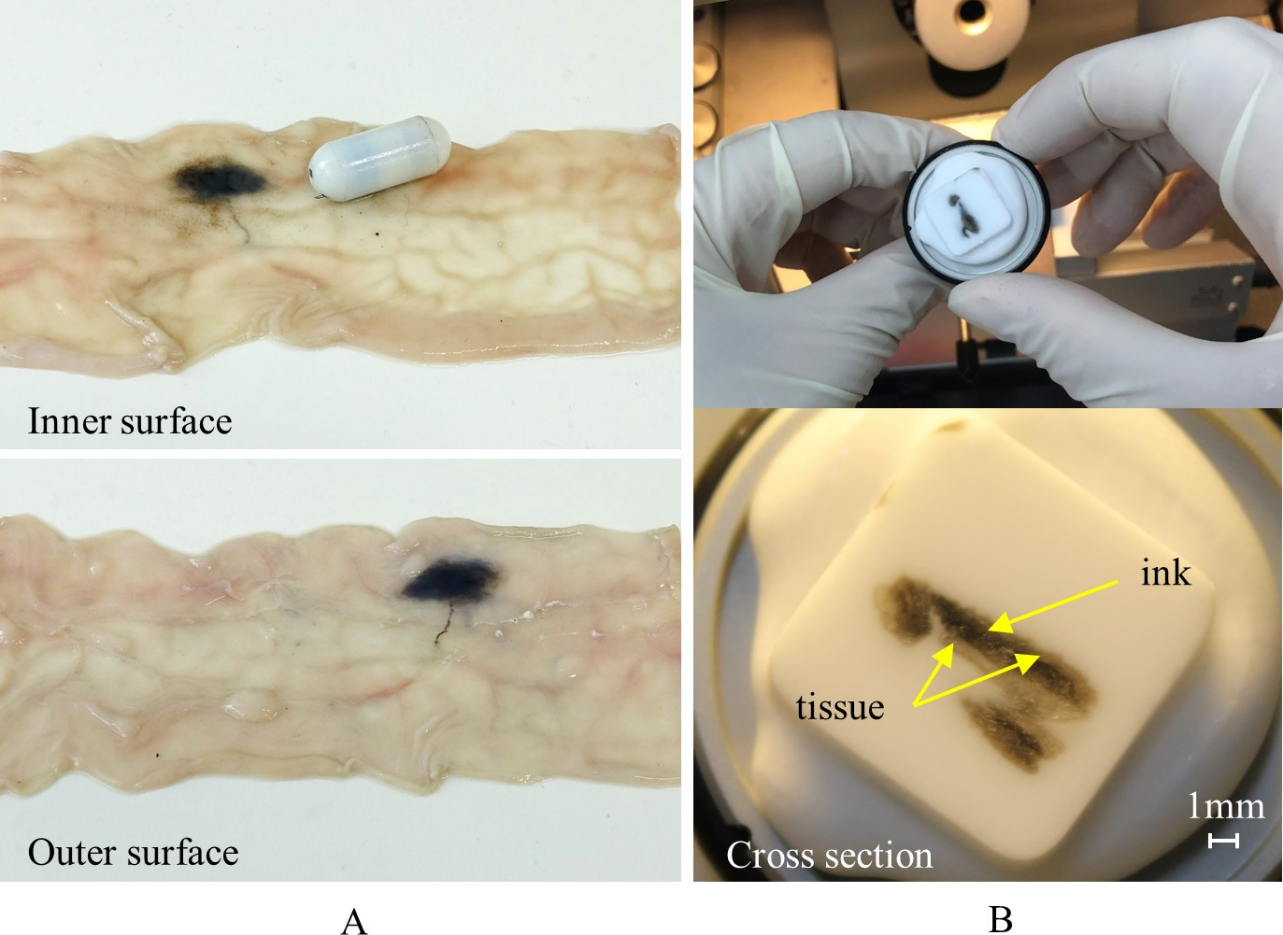
Tattooed tissue verification. (A) Inner and outer surfaces of the tattooed intestine. (B) Cross-section inspection of the dyed tissue.

The experiments were repeated five times; the TCE could move and target the desired lesion in all five experiments. Furthermore, the tattooing needle extraction and tattooing ink injection into the tissue were performed successfully through all the trials. Fig 15A shows the two sides of the tattooed small intestine 30 minutes after it was taken out and washed. The marked site is clearly visible from the outer surface that helps a specialist in laparoscopy. To verify the goodness the dyed tissue, a piece of tattooed area was sampled and fixed in 4% paraformaldehyde solution at −80°C to be solidified. The tissue was then sliced until the black dyed tissue appeared. As shown in Fig 15B, the ink was injected correctly between the tissues layers, which ensures that it can last long enough till the next diagnosis. The TCE was also tested on large intestine of pig; it was successful to mark the given target. Therefore, we confirm that the TCE executed its tattooing function successfully in both small and large intestine and the targets were localized.

## 4. Conclusions

In this study, we proposed an untethered active WCE prototype equipped with a tattooing module for preoperative laparoscopic surgery. The wireless tattooing capsule was driven by an EMA system to move and visualize the GI tract for localizing the target lesion. The external controlled EMA system achieved both flexible locomotion and tattooing ink injection by activating the chemical reaction in the TCE through the developed mechanism and control methodology. Moreover, the injection needle could be controlled externally, which can overcome the challenge of preventing damage to the organs during locomotion.

The TCE system proposed in this study is the first prototype of the wireless TCE. Moreover, the developed mechanism, which incorporates both external electromagnetic field and internal chemical gas reaction, can be used as an untethered micro/mesoscale system actuator. Thus, the proposed methodology has a potential of being applicable in the development of non-invasive medical microrobot for various other clinical purposes, such as biopsy, drug delivery, and microsurgery in intestines.

However, the proposed mechanism has visual limitation while puncturing the injection needle into tissue because of the position of camera. The thickness of the rectal mucosa varies from 0.66 to 1.13 mm, which is also a challenge for surgeons to realize the needle injection precisely. In the future, we will develop a new mechanism to increase the successful rate of needle penetration by changing the position of the needle so that the specialists can observe the injection needle while performing tattooing. Furthermore, the position tracking and localization of the developed TCE is another limitation of this study. In the future, we will integrate an advanced external localization system to the proposed TCE system [35–37], wherein the tattooing procedure can be observed and monitored for a safe and precise clinical procedure. The multiple injection mechanism is also another goal for our future work.

## Supporting information

S1 Video Tattooing capsule endoscope. (MP4)
